# Primary Amyloidosis Manifesting as Cholestatic Jaundice after Laparoscopic Cholecystectomy

**DOI:** 10.1155/2015/353818

**Published:** 2015-06-07

**Authors:** Evangelos P. Misiakos, George Bagias, Dina Tiniakos, Konstantinos Roditis, Nick Zavras, Ioannis Papanikolaou, Panagiotis Tsirigotis, Theodore Liakakos, Anastasios Machairas

**Affiliations:** ^1^3rd Department of Surgery, University of Athens School of Medicine, Attikon University Hospital, Chaidari, 12462 Athens, Greece; ^2^Department of Histology and Embryology, University of Athens School of Medicine, Goudi, 11527 Athens, Greece; ^3^2nd Department of Internal Medicine, Endoscopy Unit, University of Athens School of Medicine, Attikon University Hospital, Chaidari, 12462 Athens, Greece; ^4^2nd Department of Internal Medicine, Hematology Unit, University of Athens School of Medicine, Attikon University Hospital, Chaidari, 12462 Athens, Greece; ^5^1st Department of Surgery, University of Athens School of Medicine, Laikon Hospital, Goudi, 11527 Athens, Greece

## Abstract

A 71-year-old female patient with cholelithiasis who had undergone laparoscopic cholecystectomy was admitted with obstructive jaundice (total bilirubin ~6 mg/dL) three months later. An ERCP was performed, in which a gallstone was found, followed by a sphincterotomy and cleansing of the bile duct. Due to deterioration of jaundice (>25 mg/dL), a new, unsuccessful ERCP and stent placement was carried out. Because of ongoing cardiac failure, she underwent an echocardiogram which revealed restrictive cardiomyopathy possibly due to amyloidosis. A liver biopsy was performed, which was positive for amyloid deposits in the liver, and the diagnosis was confirmed by the detection of monoclonal *λ* IgG protein in urine. The patient's jaundice gradually deteriorated and she died one week later from hepatic insufficiency.

## 1. Introduction

The term amyloidosis describes deposits of extracellular proteins, which have common morphologic properties, affinity to certain colors, and distinctive appearance in polarized light. Amyloidosis is divided into AL and AA type. AL amyloid is derived from various regions of lambda light chain of immunoglobulin and is associated with primary amyloidosis, while the AA amyloid is derived from an acute phase protein produced in chronic inflammatory, neoplastic, or related diseases, leading to secondary amyloidosis. The most common sites of amyloid deposits are the kidneys, the heart, the peripheral nervous system, and the liver. In the liver, the clinical manifestations of amyloidosis are rare, while in primary amyloidosis, the incidence of jaundice in AL amyloidosis is less than 5%.

Herein, we present a case of primary amyloidosis with progressive obstructive jaundice, which occurred four months after a laparoscopic cholecystectomy and was finally lethal.

## 2. Case Description

A 71-year-old woman presented to our department with a 6-month history of upper right postprandial abdominal pain. She also had a history of gouty arthritis. An ultrasound of the right abdomen was carried out, which revealed gallstones and biliary sludge in the gallbladder. For that reason, an elective laparoscopic cholecystectomy was performed, without significant intra- or postoperative problems.

**Figure 1:**
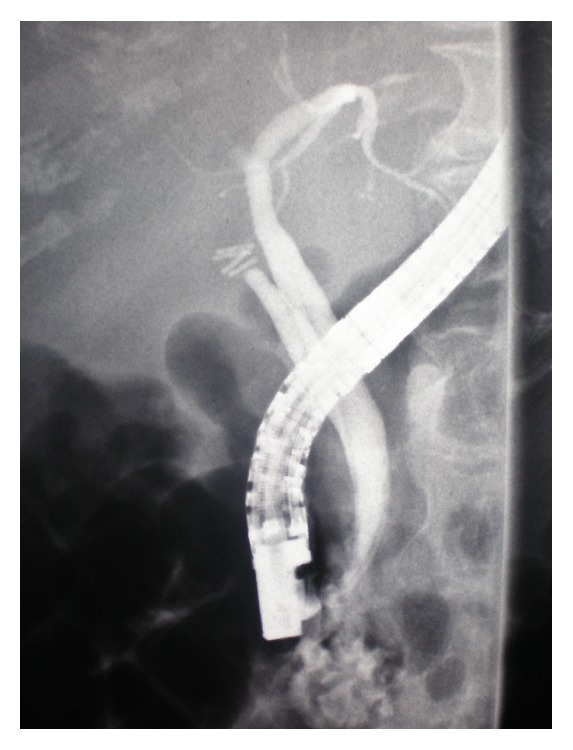
During the repeat ERCP a stent was placed in the terminal bile duct. Intrahepatic biliary ducts appear significantly narrowed.

**Figure 2:**
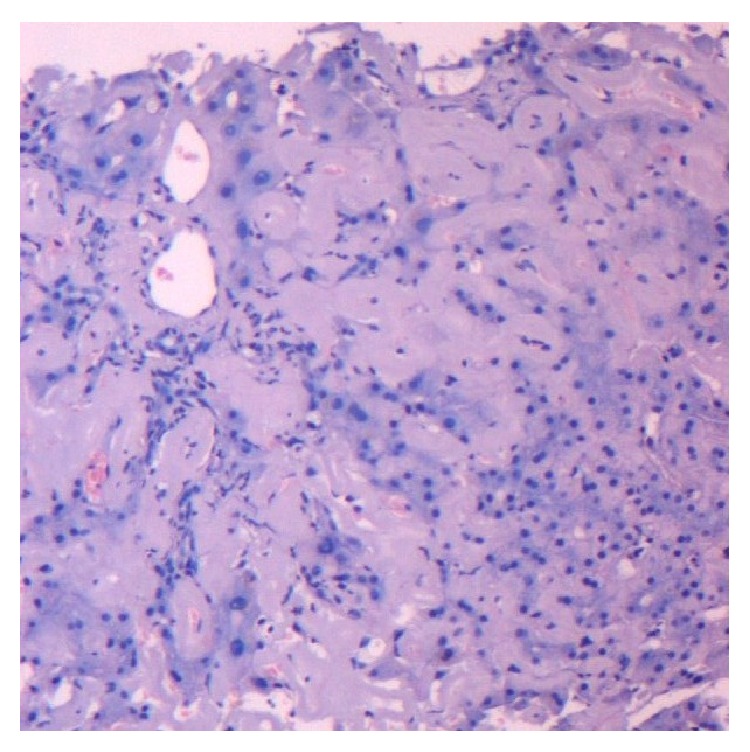
Extensive confluent accumulation of amyloid in perivascular portal tract fibrous tissue and in perisinusoidal space of Disse with associated atrophy of liver cell plates (Hematoxylin & Eosin, ×100).

After 4 months from admission, the patient presented to our department with obstructive jaundice and elevated liver function tests (serum bilirubin 7.73 mg/dL, *γ*-GT 876.00 U/L). The patient had significant hepatomegaly, without dilatation of intrahepatic and extrahepatic ducts, or other pathologies from the pancreas. A cardiac ultrasonogram was undertaken, which demonstrated cardiomegaly. The jaundice was deteriorating, whereby an ERCP was carried out, in which a small stone occluding the bile duct was found. A sphincterotomy and cleansing of the bile duct with a balloon were performed, and the patient was relieved in terms of pain but had no improvement of jaundice. As a result the ERCP was repeated and a stent was placed in the terminal bile duct, to differentiate between sclerosing cholangitis and bile duct obstruction (Figure 1). Again the jaundice did not improve, and the prothrombin time was gradually increased (INR: 1, 30). A colonoscopy was conducted, which indicated a significant mucosal edema and inflammation throughout the sigmoid and the rectum. Alongside immunodiagnostic tests were done, in which the patient was found positive in antinuclear antibodies (ANA 1 : 80). A cardiac ultrasonogram was repeated, and the findings were consistent with restrictive cardiomyopathy with diastolic dysfunction, because of possible infiltration of the myocardium with amyloid. As long as the jaundice was worsening (total bilirubin 27.51 mg/dL) a transjugular liver biopsy followed, and the patient was transferred to a hepatology center. The liver biopsy was positive for amyloid deposits (Figure 2), and when the biopsy material was dyed with Congo red, the deposits appeared red, which under polarized light had a green birefringence. The diagnosis of amyloidosis was strengthened with the detection of monoclonal *λ* IgG protein in the urine, and the finding of the bone marrow specimens infiltrated with plasma cells. The patient received chemotherapy with alkylating agents (melphalan) without response and one week later presented hepatic coma and died at home.

## 3. Discussion

The most common syndromes of amyloidosis occur after infiltration of the kidneys, heart, and peripheral nervous system by amyloid. The deposition of the amyloid in the liver is also a frequent finding in patients with both AL and AA amyloidosis as it occurs in 92% of cases. However, obstructive jaundice and progressive hepatic failure associated with amyloidosis are extremely rare [1]. Data from several clinical studies indicate that the overall prevalence of jaundice in AL amyloidosis is less than 5% [2]. Given that jaundice and hepatic failure in patients with hepatic amyloidosis are rare, it is important not to exclude other possible diagnoses, such as acute drug-induced hepatitis.

Clinical manifestations of liver amyloidosis are not frequent and usually are not mentioned. Hepatomegaly is one of the leading signs in these cases [2], often causing right upper quadrant fullness and discomfort, as in our case. Moreover symptoms associated with food intake, such as early satiety, nausea, dyspepsia, and weight loss, are also noted [3]. Other signs and symptoms are relatively uncommon and mild. Ascites can be found in some patients [4], but it is usually present in patients with systemic amyloidosis (e.g., in patients with congestive heart failure or nephrotic syndrome). However, it is rarely found in hepatic amyloidosis. Splenomegaly is also a rare manifestation [2]. Autonomic or peripheral neuropathy, carpal tunnel syndrome, and gastroparesis are also very rare manifestations [5].

In our case the presence of an IgG paraprotein indicates that this was a case of primary or immunocyte-related (AL) amyloidosis. This statement is also justified by the absence of clinical features of multiple myeloma or a known chronic liver disease which could lead to secondary amyloidosis. Usually in these cases the amyloid protein is derived from light chain fragments of immunoglobulins. The occurrence of jaundice in patients with primary liver amyloidosis is difficult to explain. Some researchers have suggested that it is a result of an impedance to bile flow at the level of the intrahepatic biliary ducts, because of large quantities of amyloid in the space of Disse, which interfere with bile and blood flow resulting in cholestatic jaundice [6]. However, the deposition of the IgG paraprotein in the liver can result in atrophy, degeneration, and necrosis of the liver cells with subsequent regenerative changes, contributing to the pathological liver function tests [4].

In our case the serum bilirubin was significantly risen, although this represents a rare finding, occurring only in 4–8% of reported cases [3]. Many studies concluded that, among the biochemical markers of liver involvement, the elevated alkaline phosphatase (ALP) is the most common finding, as it is reported to be present in 16–86% of cases of amyloidosis [7]. Some authors have mentioned also decreased levels of albumin, possibly as a result of nephrotic syndrome or reduced synthesis of albumin by the affected liver [8]. Although our patient did not have low values of albumin, she had increased prothrombin time, indicating deranged hepatic function. Peters et al. [2] have pointed out the role of a low globulin level, which seems to be grounded to the suppression of normal immunoglobulin synthesis by an abnormal plasma cell clone.

Primary hepatic amyloidosis is difficult to diagnose. In our case the diagnosis was established only with liver biopsy. Ultrasound and CT scans are typically nondiagnostic, and serum and urine electrophoresis or immunofixation electrophoresis usually have a minimal contribution to diagnosis [3]. The only reliable diagnostic tool is liver biopsy. We performed a transjugular biopsy, as a percutaneous biopsy was contraindicated, because of the elevated prothrombin time (INR 1, 30). Moreover, a percutaneous biopsy could carry an increased risk of bleeding, which may possibly lead to hepatic failure [2, 9, 10].

The histopathologic findings in hepatic amyloidosis are overall architectural distortion, alterations of portal triads, portal fibrosis, and amyloid depositions in parenchyma and/or blood vessel walls. In addition, when the biopsy material is dyed with Congo red, the deposits appear pink or red, which under polarized light has a green birefringence [8]. It has also been referred that the deposits capture the Congo red variously, and the intensity of the birefringence also varies, probably due to the heterogeneity of the deposits, as they can consist of AL amyloid and nonamyloid light chains [11]. The affinity for Congo red of cases of hepatic amyloidosis has been doubted by some authors [12], but Congo red dying is more widely used nowadays than electron microscopy, which is considered to be a more sensitive diagnostic technique.

Elevated serum bilirubin is a relatively rare finding, but it is the only proven biochemical marker related to prognosis [3]. The survival time in patients with elevated serum bilirubin is between 0.5 and 15 months. Our patient had notably elevated serum bilirubin, and she survived 4 months after the first manifestation of postoperative jaundice. The mean survival of patients with primary amyloidosis is less than 2 years [13], and it becomes shorter in patients with significant hepatic involvement [12]. Congestive heart failure affects also the survival rate, as well as the coexistence of multiple myeloma [14]. The treatment options are few and not curative. Our patient was treated with high doses of melphalan, which is the most commonly used treatment option for patients with primary liver amyloidosis. Other options are high doses of dexamethasone or thalidomide [15].
